# Impact of the flour of Jerusalem artichoke on the production of methane and carbon dioxide and growth performance in calves

**DOI:** 10.14202/vetworld.2018.1532-1538

**Published:** 2018-11-03

**Authors:** Sintija Jonova, Aija Ilgaza, Inga Grinfelde, Maksims Zolovs

**Affiliations:** 1 Preclinical Institute, Latvia University of Life Sciences and Technologies, Faculty of Veterinary Medicine, Kr. Helmana Street 8, Jelgava, LV-3004, Latvia; 2 Department of Environment and Water Management, Latvia University of Life Sciences and Technologies, Faculty of Environmental and Civil Engineering, Akademijas Street 19, Jelgava, LV-3001, Latvia; 3 Department of Biosystematics, Daugavpils University, Institute of Life Sciences and Technology, Parades Street 1a, Daugavpils, LV-5401, Latvia

**Keywords:** calves, carbon dioxide, inulin, methane, weight gain

## Abstract

**Aim:**

The aim of the research was to evaluate the growth performance, to measure the amount of methane (CH_4_) and carbon dioxide (CO_2_) in calves’ rumen, and to compare the obtained results between the control group (CoG) and the experimental group (Pre12) which received the additional supplement of the prebiotic inulin.

**Materials and Methods:**

The research was conducted with ten Holstein Friesian (*Bos taurus* L.) crossbreed calves with an average age of 33±6 days. Calves were split into two groups: 5 calves that were fed with the control non-supplemented diet (CoG) and 5 calves that were fed with the same diet further supplemented with 12 g of flour of Jerusalem artichoke (*Helianthus tuberosus* L.) containing 6 g of prebiotic inulin per 0.5 kg of barley flour diet (Pre12). The duration of the experiment was 56 days. CH_4_ and CO_2_ were measured using cavity ringdown spectroscopy device Picarro G2508. The weight and samples from calves’ rumen were evaluated 3 times during the experimental period - on the 1^st^, 28^th^, and 56^th^ days. Samples were obtained by puncturing the calf rumen.

**Results:**

The weight gain (kg) during the whole experimental period was higher in the Pre12 (65.8±6.57) compared to CoG (36.8±7.98) calves (p<0.001). The daily weight gain was also increased in the Pre12 (1.2±0.12) than CoG (0.7±0.14) calves (p<0.001). There was no difference in the levels of CH_4_ and CO_2_ produced in the rumen of CoG and Pre12 calves (p>0.05).

**Conclusion:**

The main results showed that the prebiotic inulin can promote weight gain in calves, without affecting the mean concentration of CH_4_ and CO_2_ in calves’ rumen.

## Introduction

Global climate changes are primarily caused by greenhouse gas (GHG) emissions that result in warming of the atmosphere [1]. Anthropogenic (human-caused) GHG emissions since the pre-industrial era have dramatically risen, mostly because of carbon dioxide (CO_2_), methane (CH_4_), and nitrous oxide (N_2_O). For reducing and managing the negative climate changes, both strategies of adaptation and mitigation are important. Substantial reduction of emissions over the next few decades can reduce the negative climate changes in this century and in the following centuries [2]. World population will grow from 7.5 billion today to 9.7 billion, in 2050. The requirement for animal products such as meat and milk, in 2050, is predicted to grow by 73% and 58%, respectively, compared to their levels, in 2010. Nowadays, agriculture plays a very important role in global warming phenomena. This growing need for products is impacted by such factors as the amount of natural recourses, including land, water, and nutrients since they are continually reduced. To slow down this process, waste and GHG emissions must be reduced [3]. The largest driver of global warming is CO_2_ and the second contributor to global warming after CO_2_ is CH_4_ and accounts for 16% of all GHG emissions [4]. The global warming potential of CH_4_ is 21 times more than CO_2_ [5].

Animal husbandry is a significant source of GHG emissions worldwide. The livestock sector is responsible for 14.5% of global GHG emissions. The primary livestock GHG emissions are CO_2_, CH_4_, and N_2_O. CH_4_ contributes the most to livestock GHG emissions (44%), followed by N_2_O (29%) and CO_2_ (27%) [1]. GHG emissions from cattle represent about 65% of the livestock sector emissions, making cattle the largest contributor to total sector emissions [3].

In general, the emitted amount of CO_2_ from livestock during the respiration process is not considered to be a source of CO_2_ emissions because they are part of the global biological system cycle. The vegetation consumed by the animal originates from the conversion of atmospheric CO_2_ to organic compounds or biomass. Therefore, it is assumed that the consumed amounts of CO_2_ in vegetative form are equivalent to those emitted by the livestock. Contrary, the animal is a carbon sink because the consumed carbon absorbed into live tissues of the animal and in its products, such as milk [6].

However, the situation it is not the same with CH_4_. Ruminants (cattle, sheep, goat, and others) produce CH_4_ as part of their digestive process [3]. There are billions of microorganisms, including bacteria, *Archaea* (CH_4_-producing microorganisms), protozoa, and fungi living in the rumen. These microorganisms convert carbohydrates into simple molecules that can be digested by the animal and produce volatile fatty acids (VFAs), CO_2_, ammonia, and CH_4_. The VFAs are used by animal as an energy source, but gases are removed mainly through the mouth by belching [7]. CH_4_ is the final product in anaerobic microbial fermentation processes in the gastrointestinal tract of ruminants particularly in the rumen [8]. During this fermentation, hydrogen is released and is removed through the activity of CH_4_-producing microorganisms that they unite CO_2_ with hydrogen to form CH_4_. Hence, CH_4_ emissions provide a mechanism for preventing the accumulation of hydrogen in the rumen that can negatively impact animal productivity. Therefore, when thinking about strategies for reducing the CH_4_ emissions, we have to provide an alternative way to remove hydrogen [9].

The CH_4_ mitigation from cattle has an economic as well as environmental benefits [7]. The most promising approach for reducing CH_4_ emissions from livestock is by improving the productivity and efficiency of livestock production so that less CH_4_ is produced per unit of meat or milk [10]. The other way is to directly modify the rumen fermentation so that less CH_4_ is produced [7].

The majority of CH_4_ emissions occur in ruminant herds with low productivity. Part of the mitigation potential can be achieved through the improvement of animal and herd efficiency [3]. Improvement of animal productivity and therefore minimizing GHG production per unit of derived product have become the focus of most researchers [2,11].

Changes in animal diet and addition of different feed additives have been identified as the main strategies for the mitigation of CH_4_ production. Higher emissions in ruminants are related with low feed digestibility (that results in higher enteric and manure emissions), poorer animal husbandry, lower slaughter weights (slow growth rates lead to increased emissions per kg of meat produced), or greater age at slaughter (longer life also leads to increased emissions) [3].

There are several feed supplements that have been examined by many authors and appear to have the potential to reduce the production of CH_4_ in ruminants and increase the weight gain. The majority of prebiotic substances identified till today are fermentable carbohydrates that are usually nondigestible, nonabsorbable in the small intestine, readily fermented by the beneficial gut microbiota, and poorly fermented by potentially pathogenic bacteria in the gut and mouth [12]. Dietary fibers that have proven to be beneficial prebiotic supplements include pectin, oat gum, sugar gum, resistant starch, and nondigestible oligosaccharides such as fructooligosaccharides, galactooligosaccharides, transoligosaccharides, polyfructant inulin, and lactulose [13]. Many studies exist concerning different prebiotics and their impact on CH_4_ emission and weight gain in calves. For example, the addition of galactooligosaccharides to dairy cattle diet causes the reduction of CH_4_ production up to 11% [14]. In some studies with calves, supplementation with mannan-oligosaccharides has been shown to increase average daily weight gain and feed efficiency [15,16].

Nowadays, animal scientists are exploring the efficiency of prebiotic inulin for modulating the gut ecosystem of both ruminants and non-ruminants. It is already discovered that prebiotic inulin can desirably change gut ecosystem. As indicated, inulin prebiotic reduces rumen ammonia nitrogen and CH_4_ production and increases the synthesis of microbial protein and live weight gain in calves [17-19].

Since there are just a few studies implemented about the prebiotic inulin and its impact on live weight gain and CH_4_ emission in calves and the results of our previous research were not completely convincing, the aim of this research was to measure the amount of CH_4_ and CO_2_ in calves’ rumen and also compare the live weight gain between the control group (CoG) and the experimental group which received the additional supplement of the prebiotic inulin in a recommended dosage that coincided with the best weight gain in calves’ from our previous study. Mitigating CH_4_ losses from cattle has economic as well as environmental benefits.

## Materials and Methods

### Ethical approval

All procedures performed in studies involving animals were in accordance with the ethical standards. The study was approved by the Animal Welfare and Ethical Council of the Faculty of Veterinary Medicine, Latvia University of Life Sciences and Technologies (protocol no. 2017/2).

In our experiment, the method of collection of rumen fluid was invasive (puncturing the abdomen). This caused some pain in calves; moreover, we needed to slaughter calves at the end of the experiment to obtain samples from the gastrointestinal canal for histological examination. Following the ethical requirements to minimize the number of animals used in experiments, we chose to organize as small groups as possible (five animals in each group).

### Animals

The research was conducted in the dairy cow farm in Latvia, Saldus District. The research was performed from December 2017 to April 2018. Ten clinically healthy randomly selected Holstein Friesian and Red Holstein (*Bos taurus* L.) crossbreed calves of mean age 33±5 days, and initial body weight of 74.4±10.42 kg were used in the present study. All calves were housed in groups in a pen with three solid sides and one partly open. There was enough space for all the animals to lie in comfort at the same time, move around freely, and interact with each other. The floor was concrete and was covered with a thin layer of straw as bedding. This space was equipped with two feed bunks, one for fodder and other for milk replacer. The space requirements of each pen were suitable for eight calves, but during this study, no more than five calves were housed in it. We carefully monitored the health status of calves during the whole experimental period and a month after its completion of the experiment.

After birth, all calves received the same feed: The first meal was colostrum, and for the next 5 days, calves received whole milk (3.5 L twice/day), and later the milk replacer in a dosage appropriate to their age and weight. Calves from 4 to 8 weeks of age received 8 L of milk replacer per calf/day and a prestarter without restriction (if calculated then around 0.5 kg/calf/day). From 8 to 12 weeks of age, calves received approximately 1.5 kg of barley flour and 6 L of milk replacer/calf/day. During the whole experimental period, calves had free access to an unlimited amount of hay and water. The nutrient content of all feeds is given in Table-1.

**Table-1:** Nutrients content per kg DM of diets used during calf rearing.

Item	Unit	Prestarter*	Milk replacer**	Hay	Barley flour
Crude protein	%	18.0	23.5	9.3	11.8
Fat	%	5.2	12.5	2.4	2.7
Crude fiber	%	7.0	3.5	32.1	3.3
Crude ash	%	6.5	9.0	5.6	4.4
Calcium	g/kg	14	9	3.3	4.4
Phosphorus	g/kg	7	7	3	4
Sodium	g/kg	6	4	0.2	16.2

* Each kilogram of diet contains: Vitamin A, 35.000 IU; Vitamin D3, 3.000 IU; Vitamin E, 250 mg; Vitamin B1, 13 mg; Vitamin B2, 6 mg; Vitamin B6, 5 mg; Vitamin B12, 40 mcg, niacin, 36 mg; Ca-D-pantothenate, 20 mg; folic acid, 2 mg; Vitamin K3, 2 mg; choline chloride, 500 mg; biotin 1.000 mcg; Fe, 180 mg; Cu, 25 mg; Se, 0.4 mg; Zn, 120 mg; Mn, 120 mg, I, 3 mg; Co, 1 mg.

** Each kilogram of diet contains: Vitamin A, 48.000 IU; Vitamin D3, 4.000 IU; Vitamin E, 100 mg; Vitamin B1, 16 mg; Vitamin B2, 8 mg; Vitamin B6, 6 mg; Vitamin B12, 50 mcg, niacin, 50 mg; Vitamin C, 250 mg; Ca-D-pantothenate, 25 mg; folic acid, 1 mg; Vitamin K3, 2 mg; choline chloride, 300 mg; biotin 200 mcg; Fe, 100 mg; Zn, 36 mg; Cu, 4 mg; Se, 0.4 mg; Mn, 48 mg, I, 1 mg. DM=Dry matter

### Experimental design

Calves were allocated into two groups: 5 calves in the CoG and 5 calves that were further supplemented with 12 g of flour of Jerusalem artichoke (*Helianthus tuberosus* L.) per head containing 6 g of prebiotic inulin (per 0.5 kg of barley flour diet) (Pre12). The prebiotic was added to barley flour once a day in the morning.

Jerusalem artichoke contains just around 10% of inulin, but using special technologies, the content of inulin can be increased up to 48.5-50.1% out of the dry weight [18,20,21]. The supplement containing prebiotic inulin which is used in this study is produced in Latvia at the University of Latvia Institute of Microbiology and Biotechnology.

The length of the experiment was 8 weeks (56 days). CH_4_ and CO_2_ were measured using cavity ringdown spectroscopy device Picarro G2508, manufactured by company “Picarro,” The United States of America. The samples from calves’ rumen were evaluated 3 times during the research with an interval of 4 weeks - on the 1^st^, 28^th^, and 56^th^ days of the research. Samples were obtained by puncturing the calf rumen 2 h after feeding with 16-G needle and were collected into 20 ml syringes. The site of puncturing was the upper left flank where visually was noticed the accumulation of gases. After collection, 10 ml of its gas sample was injected into the gas analyzer and measured for 180 s.

The weight of calves was determined using special weight measuring tape Animeter. The weight can be defined by measuring the girth of the chest immediately behind the elbows.

### Statistical analysis

The assumption of normal data distribution was assessed by Shapiro–Wilk’s test and visual inspection of their histograms and normal Q-Q plots. The assumption of homogeneity of variances was tested by Levene’s test. Box plots detected the presence of three outliers that were removed from the analysis. To determine whether there are any statistically significant differences between three or more independent groups, we used Kruskal–Wallis H test with pairwise comparisons using Dunn’s procedure [22] with a Bonferroni adjustment. To determine whether there are any statistically significant differences between two groups, we used Mann–Whitney U-test or independent samples t-test. The association between calves weight and concentration of CO_2_ and CH_4_ in calves’ rumen was evaluated by Pearson correlation. Those tests were carried out using SPSS statistics version 22 (IBM Corporation, Chicago, Illinois). All statistical analyses were performed at the significance level of α=0.05.

## Results

The weight gain of calves between the 1^st^ and 28^th^ days, between the 28^th^ and 56^th^ days, and between the 1^st^ and 56^th^ days of the experimental period is presented in Table-2. The data of initial and daily live weight gain were normally distributed (p>0.05), and there was the homogeneity of variances (p>0.05). Independent samples t-test showed that the weight gain during the whole research was higher in the Pre12 (65.8±6.57) than CoG calves (36.8±7.98) (p<0.001). Furthermore, the daily weight gain was significantly increased in the Pre12 (1.2±0.12) than CoG calves (0.7±0.14) (p<0.001).

**Table-2:** Growth performance of calves.

Parameters	Group		p-values

	CoG	Pre12	
Initial mean live weight (kg±SD)	79.4±10.53	69.4±8.41	0.056
Mean live weight gain (kg±SD and %):			
1^st^-28^th^ research days	17.6±4.93 (+22.2)	22±4.42 (+31.7)	0.288
28^th^-56^th^ research days	19.2±4.97 (+19.8)	43.8±6.91 (+47.9)	0.001*
1^st^-56^th^ research days	36.8±7.98 (+46.3)	65.8±6.57 (+94.8)	0.001*
Final mean live weight (kg±SD)	116.2±16.30	135.2±14.32	0.056
Mean daily weight gain (kg/day)	0.7±0.14	1.2±0.12	0.001*

* Significant at p<0.05. CoG=Control group

The data of CO_2_ and CH_4_ concentration in calves’ rumen during the 1^st^, 28^th^, and 56^th^ days are presented in Table-3. The data of CO_2_ and CH_4_ were not normally distributed (p<0.05), and Mann–Whitney U-test showed that there were no differences in mean rumen levels of CH_4_ and CO_2_ between CoG and Pre12 calves (p>0.05). Furthermore, Kruskal–Wallis H test showed that there was no difference in concentration of CH_4_ and CO_2_ in calves’ rumen among the different sampling days of the experiment (p>0.05). There was a moderate positive correlation between calves weight and concentration of CO_2_ r (30)=0.421, p=0.021, and no correlation between calves weight and concentration of CH_4_ (p>0.05).

**Table-3:** The mean amount of CH_4_ (mg/m^3^±SD) and CO_2_ (mg/m^3^±SD) in calves’ rumen during the experiment.

Day of the experiment	Gas	Group	

		CoG	Pre12
1^st^ day of the experiment	CH_4_	600.0±429.23	904.8±224.31
	CO_2_	3165.1±372.35	4141.2±622.55
28^th^ day of the experiment	CH_4_	1011.3±151.21	849.6±66.55
	CO_2_	4530.4±369.65	3919.3±440.45
56^th^ day of the experiment	CH_4_	863.3±164.17	893.7±213.12
	CO_2_	4260.8±250.69	4852.1±695.21

CH_4_=Methane, CO_2_=Carbon dioxide, CoG=Control group

The mean CH_4_ production in calves’ rumen per kg of body weight in CoG on the 1^st^ day of the experiment was Me=9.630 (IQR 1.171-9.856) mg/m^3^ but in Pre12 Me=12.496 (IQR 11.061-14.490) mg/m^3^. On the 28^th^ day of the experiment in CoG, the amount of CH_4_ on per kg of body weight increased to Me=10.334 (IQR 9.551-10.901) mg/m^3^ but in Pre12 group decreased - Me=9.141(IQR 8.911-9.195) mg/m^3^. At the end of the experiment, on day 56^th^, the amount of CH_4_ on 1 kg body weight in CoG had reached the lowest level during the whole experimental period Me=7.092 (IQR 6.350-9.188) mg/m^3^. The same trend was observed in the Pre12 group - Me=5.489(IQR 4.994-8.761) mg/m^3^.

The mean CO_2_ production in calves’ rumen calculated on 1 kg body weight in CoG on the 1^st^ day of the experiment was Me=38.355 (IQR 36.319-39.258) mg/m^3^ and in Pre12 Me=51.884 (IQR 50.228-63.863) mg/m^3^. On the next sampling day, the amount of CO_2_ on 1 kg body weight in CoG had increased Me=46.420 (IQR 42.583-48.605) mg/m^3^ but in Pre12 group decreased - Me=43.574 (IQR 35.703-45.931) mg/m^3^. On the 56^th^ day of the experiment in Co group, this amount of CO_2_ gas had reached almost the same amount as on the 1^st^ day - Me=36.724 (IQR 34.628-37.074) mg/m^3^, but in Pre12 group, the amount of CO_2_ on 1 kg body weight continued to decrease and had reached the level of Me=33.098 (IQR 32.541-43.388) mg/m^3^.

## Discussion

Prebiotic supplementation at known dosages might provide significant advances to ruminant health and production [12]. The main results of our experiment showed that the prebiotic inulin added to barley flour once a day in the mornings can promote rapid weight gain in calves.

There are several studies which show the positive effect of inulin on calves’ growth performance. Król [23] performed an experiment with 36 Holstein Friesian calves from 1^st^ to 56^th^ days of their life. He allocated animals into three groups (12 calves in each): A CoG, a group that received 3 g of inulin/day/head and a group that received 6 g of inulin/day/head. He observed that calves that received inulin at the level of 6 g/day/head showed clearly (p≤0.01; p≤0.05) higher final body weight than animals which received this prebiotic at the level of 3 g/day/head and animals that did not receive inulin at all.

In our study, we noticed a significant difference (p<0.001) in live weight gain between the CoG and the group of calves which received 12 g of the flour of Jerusalem artichoke containing 6 g of prebiotic inulin. Higher live weight gain was noticed in the group which received the feed additive. Similar results were observed by Ārne and Ilgaža [17] in a study on male Holstein Friesian calves with an age of 23±5 days and with an initial weight of 50±5 kg after the addition of the flour of Jerusalem artichoke containing 48.5-50.1% of inulin at different dosages to their diets. 10 calves were fed daily with 6 g of prebiotic (12 g of flour of Jerusalem artichoke), 10 calves with 12 g of prebiotic (24 g of flour of Jerusalem artichoke), 10 calves with 24 g of prebiotic (48 g of flour of Jerusalem artichoke), and 10 did not receive feed supplement. As indicated, inulin supplementation significantly increased animal live weight gain, with an optimal inulin dosage of 6 or 12 g/day/calf. Authors observed that optimal inulin dosage is 6 g/day/calf or 12 g/day/calf.

Król [23] also noticed that the highest daily body weight gain was observed in calves that were offered inulin at the level of 6 g/day/head, but differences were not statistically significant. These findings are in contrast to our results since the daily weight gain was increased in the Pre12 calves (p<0.001). Our results coincide with some other authors who concluded that inclusion of inulin in calves feed leads to significantly higher live weight gains [24-26].

These positive effects on growth performance of calves could be a result of prebiotics that are considered as a potential method for beneficially modulating the gut microbiota within the ruminant and improve animal health. In particular, prebiotics can improve overall ruminant health and performance by enhancing nutritional, digestive, and metabolic processes, growth and development, and immune system capabilities [27].

When talking about prebiotics and their positive effects on ruminants, some factors should be taken into account. The rumen of a fully grown ruminant represents a huge fermentation organ in which the dietary inulin would be completely fermented and would not reach more distal areas of the gastrointestinal tract where it can perform its beneficial activities. In calves, however, that are fed mainly a milk replacer, the rumen does not develop so fast, and from a digestive point of view, the animal could be considered as a monogastric [28].

The rumination processes in calves fully begin at about 6-8 weeks of age. At this time, the solid feed is the main source of nutrition, and the rumen comprises approximately 70% of all stomach compartments. However, calves can be considered as full ruminants at 12 weeks of age when their rumen is fully developed, and calves are able to eat and digest solid feed at the level of adult ruminants [29]. Hence, according to these facts, we can conclude that prebiotics, including inulin, have their maximal effect in ruminants at very early age, before their conversion into full ruminants. In previous literature as well as in our study, calves were in their preruminant or transitional stage of life so we believe that the greater live weight gain and daily weight gain in calves which were supplemented with additional 12 g of flour of Jerusalem artichoke containing 6 g of inulin/calf/day are a result of the prebiotic’s positive action in distal areas of the gastrointestinal tract.

There is a huge diversity of different microorganisms in the rumen including several methanogens, which are *Archae* that release CH_4_ under highly anaerobic conditions [30]. As mentioned before, CH_4_ is formed by methanogens utilizing CO_2_ and hydrogen [31]. These methanogens consume 2-15% of ingested energy from ruminants during the production of CH_4_ [8,30]. Furthermore, high levels of methanogenesis in the rumen could reduce productivity and have negative impacts on the ability of ruminants to sustain high levels of production [30]. Recent research has been devoted to characterize methanogen populations within the ruminant to find ways of reducing their adverse effects on production and the environment.

In our research, we concluded that the addition of prebiotic inulin to barley flour did not significantly impact the production of CH_4_ and CO_2_ in calves’ rumen. There are many researchers that have used different prebiotics and found a positive effect on CH_4_ reduction in ruminants. For example, Hindrichsen *et al*. [32] performed an experiment using rumen stimulation technique (RUSITEC). The study included eight diets. Every diet had a forage-to-supplement ratio of 1:1. In addition, treatment feeds included Jerusalem artichoke tubers containing inulin. The eight diets were simultaneously tested in an eight fermenter RUSITEC. The experiment consisted of four consecutive runs of 10 days each. The supplements rich in sugar (molasses, Jerusalem artichoke, and apple pulp) had a positive effect on reducing the production of CH_4_. This was even true when comparing the diets at the level of CH_4_ release per gram of degraded fiber. Sugars are likely to have a higher CH_4_-producing effect than starch and fibrous carbohydrates under the condition of a high ruminal pH, i.e., with mixed diets containing a high proportion of forage.

Although some studies have compared the effect of starch and inulin on the production of CH_4_, results remain contradictory. Czerkawski and Breckenridge [33] found *in vitro* that 1.5 g of inulin substrate produced 4.2 mL of CH_4_ in the 1^st^ h, while starch produced only 0.6 mL of CH_4_. However, Poulsen *et al*. [34] found no differences between inulin, wheat, and cornstarch in CH_4_ production rates and total CH_4_ production after 48 h. Similar results were also observed in the study by Hindrichsen *et al*. [32]. In the study by Zhao *et al*. [35], CH_4_ production was lower for inulin compared with starch. These discrepancies suggest that estimation of production of CH_4_ from inulin fermentation needs further research.

In our research, we noticed that higher CH_4_ and CO_2_ production in calves’ rumen was in the group supplemented with additional 12 g of flour of Jerusalem artichoke containing 6 g of prebiotic inulin, although these results were not significantly different from those noticed in CoG. However, still, this made us think that the prebiotic inulin could promote the production of both of these gases in calves’ rumen.

There are just a few studies about inulin and its impact on production of CH_4_ and as indicated almost all of them show that this prebiotic does not reduce the production of CH_4_ or even increase its production in ruminants. However, there are many publications about probiotics which had shown a positive effect on ruminants. For example, the addition of yeast cultures (probiotics) to the ruminant diet improved productivity in both lactating and growing animals. Yeast also can change the fermentation process in the rumen in such a way that reduces the formation of CH_4_ [36].

## Conclusion

Based on results of our research, we conclude that flour of Jerusalem artichoke at a dosage of 12 g/calf, containing 6 g of inulin, significantly improves the growth performance of calves, increases the average daily weight gain, and also increases the final live weight gain. However, inulin at this dosage does not affect the production of CH_4_ and CO_2_. To confirm our findings, further research is needed with more number of calves in the experimental groups and our further interest is also to perform a study on calves by adding a live yeast *Saccharomyces cerevisiae*, 1026 strain as this probiotic has a potential to improve growth performance and reduce CH_4_ production in ruminants.

## Authors’ Contributions

SJ collected the samples, performed the clinical examination of calves, and drafted the paper and revised it. AI designed the concept for this research and scientific paper. IG performed the analysis of rumen gases. MZ performed the statistical analysis of all data. All authors read and approved the final manuscript.
